# Low BCL9 expression inhibited ovarian epithelial malignant tumor progression by decreasing proliferation, migration, and increasing apoptosis to cancer cells

**DOI:** 10.1186/s12935-019-1009-5

**Published:** 2019-12-06

**Authors:** Jing Wang, Mingjun Zheng, Liancheng Zhu, Lu Deng, Xiao Li, Linging Gao, Caixia Wang, Huimin Wang, Juanjuan Liu, Bei Lin

**Affiliations:** 1 Department of Obstetrics and Gynaecology, Shengjing Hospital Affiliated to China Medical University, No. 36 Sanhao Street, Heping District, Shenyang, 110004 Liaoning China; 2 Key Laboratory of Maternal-Fetal Medicine of Liaoning Province and, Laboratory of Obstetrics and Gynecology of Higher Education of Liaoning Province, No. 7 Mulan Road, Xihu District, Benxi, 117000 Liaoning China; 3 Obstetrics and Gynaecology Hospital of Fudan Universuty, Shanghai, China; 4 Department of Gynecology, Liaoning Cancer Hospital & Institute China Medical University, No. 44 Xiaoheyan Road, Dadong District, Shenyang, 110000 Liaoning China

**Keywords:** Ovarian cancer, BCL9, Malignant biological behavior, Targeted therapy

## Abstract

**Background:**

Abnormal activation of the classic Wnt signaling pathway is closely related to the occurrence of epithelial cancers. B-cell lymphoma 9 (BCL9), a transcription factor, is a novel oncogene discovered in the classic Wnt pathway and promotes the occurrence and development of various tumors. Ovarian cancer is the gynecological malignant tumor with the highest mortality because it is difficult to diagnose early, and easy to relapse and metastasis. The expression and role of BCL9 in epithelial ovarian cancer (EOC) have not been studied. Thus, in this research, we aimed to investigate the expression and clinical significance of BCL9 in EOC tissues and its effect on the malignant biological behavior of human ovarian cancer cells.

**Methods:**

We detect the expression of BCL9 in ovarian epithelial tumor tissues and normal ovarian tissues using immunohistochemistry and analyzed the relationship between it and clinicopathological parameters and patient prognosis. The expression of proteins was detected by Western blot. The MTT assay, flow cytometry, the scratch assay, and the transwell assay were used to detect cell proliferation, apoptosis, migration, and invasion, respectively. A total of 374 ovarian cancer tissue samples were collected using TCGA database. A gene set enrichment analysis of BCL9 was performed.

**Results:**

BCL9 was overexpressed in EOC tissues. The level of BCL9 expression was correlated with the 5-year progression-free survival rate and overall survival rate in ovarian cancer patients and independently predicted the risk of ovarian cancer recurrence. Low BCL9 expression inhibited proliferation, invasion and migration of EOC cells, decreased MMP2 and MMP9 expression of ES-2 cell line, increased the BAX/BCL2 ratio and promoted apoptosis of EOC cells.

**Conclusion:**

BCL9 is overexpressed in epithelial ovarian tumors, resulting in a poor prognosis for ovarian cancer patients. Low BCL9 expression can promote ovarian cancer cell apoptosis, inhibit proliferation and migration. BCL9 promotes the development of ovarian cancer.

## Background

Ovarian cancer is a common malignant tumor in the female reproductive system and is associated with the highest mortality among gynecologic malignancies [1]. Most ovarian cancer cases are diagnosed only at an advanced stage, and it is extremely prone to recurrence and metastasis. Therefore, the mechanism of the occurrence and development of ovarian cancer needs to be explored to discover potential therapeutic targets for improving prognosis in ovarian cancer patients. B-cell lymphoma 9 (BCL9) is a novel oncogene in the Wnt pathway. It was first discovered by Willis in 1998 in a patient with pre-B-cell acute lymphoblastic leukemia [2]. The BCL9 gene is located on chromosome 1q21 (1; 14) (q21; q23). This chromosomal translocation causes the uncontrolled expression of BCL9, which becomes a vital part of the pathogenesis of malignant tumors. BCL9 protein comprises 1394 amino acids [2] and three conserved regions: β-catenin binding domain HD1 [3], Pygopus binding domain HD2 [4], and typical nuclear localization signal peptide HD3 [5]. BCL9 is an essential member of the Wnt pathway that can become abnormally activated. BCL9 can form a complex with stable β-catenin, pygopus family coactivator, and T cell factor transcriptional cofactor (TCF)/lymphocyte enhancer (LEF), mainly in the nucleus. This complex activates transcription and expression by binding to the promoters of many target genes [3, 5–9]. The resulting product leads to the substantial and rapid proliferation of tumor cells. Therefore, the BCL9 gene may be associated with the development and prognosis of various human malignancies. Some small-molecule inhibitors that target the interface between BCL9 and β-catenin have been extensively studied recently in colorectal cancer cells, which has provided hope to the clinical treatment of malignant tumors. However, the expression and mechanism of BCL9 in human ovarian epithelial tumor tissues are still unknown. Therefore, the present study explored the expression of BCL9 in human ovarian epithelial tumors and its relationship to clinicopathological parameters and changes in the malignant biological behavior of human ovarian cancer cells. We sought to provide a theoretical basis for further investigations of the occurrence and development of ovarian cancer and the search for new therapeutic targets.

## Materials and methods

### Specimen source

Approved by the Ethics Review Committee of China Medical University (the ethical approval code is 2010PS84 K), a total of 99 cases of surgical resection from the Department of Obstetrics and Gynecology, Shengjing Hospital affiliated to China Medical University from 2008 to 2013 were collected and made into paraffin specimens, in which normal ovarian tissues were from postmenopausal patients or cervical cancer patients with Double adnexal hysterectomy. All patients were newly diagnosed and not treated radiotherapy or chemotherapy before surgical operation. The clinical data and follow-up (more than 60 months follow-up) of patient were complete. The 99 specimens including 61 cases of ovarian epithelia malignant tumors, 13 borderline, 13 benign, and 12 normal ovarian tissues were 58.5 (25–77), 49 (19–84), 52 (36–78), and 50 (43–59) years old, respectively. There was no difference in age composition between each group (*P *> 0.05). There were 39 poor differentiation and 22 high or medium differentiation in 61 ovarian epithelia malignant tumors according to the classification criteria established by the WHO in 2009. Surgical pathological staging was performed according to the criteria established by FIGO: 24 cases in stage I–II and 37 cases in stage III–IV. There were 13 cases with lymph node metastasis, 35 cases without metastasis, and 13 cases did not undergo lymph node screening surgery.

### Immunohistochemistry

All ovarian cancer tissue samples were made into 5 μm continuous paraffin sections and dewaxed with xylene and ethanol. The samples were heated in a microwave oven and cooled to room temperature to repair the antigens. The sections were incubated in H_2_O_2_ for 30 min at 37 °C to block endogenous peroxidase. After rinsed in PBS for three times, the samples were incubated in goat serum for 20 min at 37 °C and then in rabbit anti-BCL9 (Sigma, 1:75, USA) overnight at 4 °C. The second day, the sections were rinsed in PBS for three times, incubated in horseradish peroxidase-labeled goat anti-rabbit secondary antibody at 37 °C for 30 min, and stained by 3,3′-diaminobenzidine. Using PBS to rinse the sections for three times and observed the sections which incubated in DAB (MXB, China) for 3 min by microscope. The cell nucleus was re-stained into blue by hematoxylin. Sections were dehydrated in ethanol and xylene, and covered with neutral gum. BCL9 expression in colorectal cancer tissue was used as a positive control and in the sample which incubated with PBS instead of BCL9 primary antibody were used as a negative control.

### Result determination

The results of immunohistochemical staining were determined by two observers, and a third observer arbitrated when two observers’ opinions were not consistent. The observers were unaware of the clinical datas of patients. It was considered positive if the nucleus or cytoplasm of cells were stained with brownish yellow. According to the staining intensity, it was divided into non-staining 0 points, light yellow 1 point, brown yellow 2 points, and dark brown 3 points. The percentage of stained cells was averaged from five randomly fields of sections under the 400-fold magnification microscope. All sections were scored 0, 1, 2, 3, or 4 according to the positive cell percentage that was < 5%, 5–25%, 26–50%, 51–75%, and > 75%, respectively. The final score was the product of the above two score. 0–2 were negative (−), 3–4 were weakly positive (+), 5–8 were moderately positive (++), and 9–12 were strongly positive (+++), (−) and (+) were low expression, (++) and (+++) were high expression.

### Cell culture and transfection

Both human ovarian serous adenocarcinoma cell line Caov-3 and human ovarian clear cancer cell line ES-2 were purchased from Shanghai Cell Bank of Chinese Academy of Sciences and cultured in a conditional incubator with 37 °C, 5% CO_2_, and saturated humidity. Caov-3, Skov-3 and Ovcar-3 were cultured in RPMI l640 medium and ES-2 was cultured in Mc Coys 5A medium, both medium with 10% fetal bovine serum, respectively. Cells were seeded in 6-well culture plates after digested using 0.25% trypsin with 0.02% EDTA on the day before transfection. After overnight cultured, the cell confluence reached about 50%, and transfection was performed according to the instructions of Lipofectamine 3000 (Thermo, USA). The mRNA target sequences of BCL9-siRNA were GAU CCC UCC AAA CCA UAA ATT for BCL9-siRNA1, CCC UCU AGU ACA CCU UAU ATT for BCL9-siRNA2 and GGG CAU UAA UAC ACA GAA UTT for BCL9-siRNA3. Cells were cultured using RPMI 1640 medium without antibiotics and serum. The serum-containing complete medium was replaced 6 h after transfection. The transcription and expression of BCL9 gene were detected after 48 h of transfection.

### Immunocytofluorescence

The cells were plated on circular coverslips, cultured for 24 h, and then were fixed with 4% concentration of paraformaldehyde. Then, the circular coverslips were washed three times with PBS and were incubated in H_2_O_2_ for 30 min at 37 °C to block endogenous peroxidase. After rinsed in PBS for three times, the samples were incubated in goat serum for 20 min at 37 °C and then in rabbit anti-BCL9 primary antibody (Sigma, rabbit polyclonal, 1:500, USA) overnight at 4 °C. The 2nd day, the sections were rinsed in PBS for three times, incubated in fluorescence-labeled goat anti-rabbit secondary antibody at 37 °C for 2 h. After this, the circular coverslips were incubated in DAPI for 10 min at room temperature and washed three times with PBS. Fluorescence microscopy was used to observe the localization of BCL9 in cells.

### Western blot

Total protein samples extracted from cells using RIPA buffer, were separated by 10% SDS-PAGE. After the electrophoresis, the proteins completely separated in the separation gel are transferred to the PVDF membrane. After blocked with 5% skim milk for 2 h at room temperature, the membranes were incubated in primary antibody BCL9 primary antibody (Sigma, rabbit polyclonal, 1:500, USA), PCNA primary antibody (CST, mouse monoclonal, 1:1000, USA), BCL2 primary antibody (CST, mouse monoclonal, 1:2000, USA), BAX primary antibody (CST, mouse monoclonal, 1:4000, USA), MMP2 primary antibody (Proteintech, rabbit polyclonal, 1:1000, China), MMP9 primary antibody (Proteintech, rabbit polyclonal, 1:1000, China), β-catenin primary antibody (CST, rabbit polyclonal, 1:500, USA), GAPDH primary antibody (ZSGB-BIO, mouse monoclonal, 1:3000, China) overnight at 4 °C. The 2nd day, the membrane was washed with TBST for three times, 10 min each time, and incubated in secondary antibody for 2 h at room temperature. Analyzed the protein bands that were obtained using the chemiluminescent agent.

### MTT assay

Using MTT assay to assess the proliferation of cells. 3 × 10^3^ cells were plated in per well of the 96-well cell culture plates and transfected after 24 h. Added the 20 μl of 5 mg/ml MTT into per well, mixed and incubated them for 4 h at 37 °C after the cells were transfected for 0 h, 24 h, 48 h and 72 h. Then added 150 μL of DMSO into per well to stop the reaction. The plate was measured on the absorbance of 490 nm of the plate reader (Bio-Tek, ELX808 IU, USA). Repeated the assay at least three times independently.

### Wound healing assay

Cell migration was assessed by wound healing assay. 3 × 10^5^ cells were seeded in 6 well plate the day before being transfected. A sterile 1 ml pipette tip was used to perform a scratch wound on the surface of the monolayer when the transfected cells were 90% density. Then the cells with wound were incubated in medium containing 2% serum for 24 h. We took the images of the cell wounds under the microscope. Cell migration ability was analyzed by comparing the cell scratch wound width with control group cells.

### Transwell assay

Cell invasion was assessed by transwell assay. Using serum-free medium to dilute the matrigel (BD Biosciences, NJ, USA) by 1:8, added 80 μl diluted matrigel into the upper chambers which on the 24-well culture plates and put the plates in the incubator for 30 min. 2 × 10^4^ cells were added to the upper layer of each chamber in a volume of 200 μl, and 500 μl of medium with 10% fetal bovine serum was added to the lower chamber. After culturing for 48 h, the cells were fixed in paraformaldehyde for 20 min. After rinsed in PBS for three times, the chamber was soaked in 0.1% crystal violet for 20 min to be dyed. Then, the chambers were rinsed with PBS for three times and the cells on the upper layer of polycarbonate membrane were gently wiped off with a cotton swab. Under a microscope, cell invasion was evaluated by comparing the number of invasive cells among each group. Repeat the experiment three times independently.

### Cell cycle analysis

1 × 10^6^ cells transfected for 48 h were collected and fixed in pre-cold 70% ethanol overnight. The next day, ethanol was washed away with PBS. 100 μl RNaseA (KeyGEN BioTECH, KGA511-KGA512) was added to the cells and incubated at 37 °C for 30 min. Then, propidium iodide (KeyGEN BioTECH, KGA511-KGA512) was added and incubated at 4 °C for 30 min in the dark. Using flow cytometry (BD; FACSAria, USA) to analysis the cell samples immediately. The experiment was repeated in triplicate independently.

### Annexin V/propidium iodide (PI) double staining assay

Annexin V/PI double staining assay was used to detected cell apoptosis rate. 1 × 10^5^ cells transfected for 72 h were collected and washed twice with PBS. We resuspended cells with 500 µl of binding buffer, and added 3 µl PI and 3 µl Annexin V (DojinDo, AD11) into the cells. Cell apoptosis rate was detected by flow cytometry (BD; FACSAria, USA).

### Function analysis and pathway enrichment analysis

Proteins that interact with BCL9 were found in the cBioportal database (http://www.cbioportal.org/), and the top 200 significantly related molecules were screened based on *P*-values. *P*-value < 0.05 was considered statistically significant. Gene Ontology (GO) functional analysis of genes corresponding to the above proteins was performed using DAVID Bioinformatics Resources (http://david.abcc.ncifcrf.gov/). The significance of the above gene enrichment to the Kyoto Encyclopedia of Genes and Genomes (KEGG) pathway was calculated by hypergeometric test. The hypergeometric test formula is as follows:

1 $$ P = 1 - \mathop \sum \limits_{{{\text{k}} = 0}}^{\text{m}} \frac{{\left( {\begin{array}{*{20}c} {\text{n}} \\ {\text{k}} \\ \end{array} } \right)\left( {\begin{array}{*{20}c} {{\text{N}} - {\text{n}}} \\ {{\text{M}} - {\text{k}}} \\ \end{array} } \right)}}{{\left( {\begin{array}{*{20}c} {\text{N}} \\ {\text{M}} \\ \end{array} } \right)}} $$

In formula (1), N is the number of genome-wide genes. M is the number of genes annotated to a given pathway in the whole genome. n is the number of genes in the interaction network, and m is the number of genes annotated to a given pathway.

### Data collection from TCGA database

The ovarian cancer data set was downloaded and pre-processed from the TCGA database (https://tcga-data.nci.nih.gov/tcga/) and a total of 374 tumor tissue samples were included in the study. According to the expression profile data, the BCL9 gene expression was ranked from low to high, and the samples were equally divided into four. The first 25% of the samples constitute the BCL9 low expression group. The last 25% of the samples formed a high expression group.

### Gene set enrichment analysis (GSEA)

Analysis was performed using GSEA version 3.0 software [10]. The c2.cp.kegg.v6.1.symbols.gmt data set was downloaded from the MsigDB database on the GSEA website. The sorted expression spectrum data and the attribute file were subjected to enrichment analysis by default weighted enrichment statistics. Random assortment times were set to 1000.

### Statistics

Statistical analysis of the data was performed using SPSS 21.0 and Graphpad Prism 7.0 programs. Counting data was analyzed using a χ^2^ test and Fisher exact test. Measurement data was analyzed using a t test. Using Kaplan–Meier and Log-rank analysis to analyze the survival curves of patients and a Cox model to analyze relationships between clinical data and patients. For GSEA analysis, gene clusters with false discovery rates (FDR) < 0.25 and *P *< 0.05 were considered as significantly enriched genes. *P*-value < 0.05 was considered to be statistically significant, in figures, *P < 0.05, **P < 0.01, ***P < 0.001.

## Results

### BCL9 was significantly upregulated in ovarian cancer

**Fig. 1:**
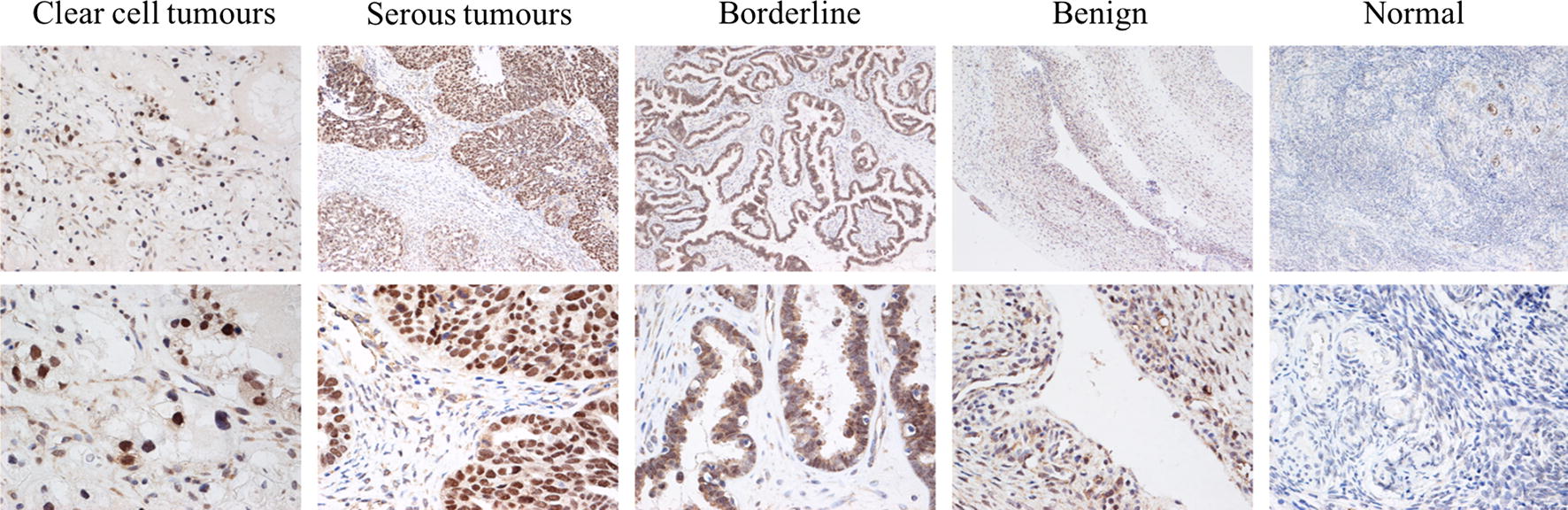
Expression of BCL9 in different ovarian tissues (upper panels: ×200 magnification; lower panels: ×400 magnification)

The immunohistochemistry results showed that BCL9 was mainly located in the nucleus in ovarian epithelial tumors and partly in the cytoplasm. The positive BCL9 expression rates in ovarian epithelial malignant, borderline, and benign tumor tissues and normal ovarian tissues were 81.97%, 76.92%, 38.46%, and 0%, respectively. The positive rate of BCL9 expression in the malignant group and borderline group was significantly higher than in the benign group and normal group. BCL9 expression was significantly higher in the benign group than in the normal group. No BCL9 expression was detected in normal ovarian tissue (Table 1, Fig. 1). No correlation was found between the level of BCL9 expression and clinical pathological parameters of ovarian cancer (Table 2).

**Table 1:** Expression of BCL9 antigen in different ovarian tissues

Group	n	BCL9				Positive cases	Positive rate %
		(−)	(+)	(++)	(+++)		
Malignant^#^	61	11	21	13	16	51	81.97
Borderline**	13	3	3	4	3	10	76.92
Benign*	13	8	4	1	0	5	38.46
Normal	12	12	0	0	0	0	0

*P < 0.05, compared with normal group

**P < 0.01, compared with normal group

^#^*P* < 0.01, compared with benign group and normal group

**Table 2:** Relationship between the expression of BCL9 and clinicopathological parameters of epithelial ovarian cancer

Feature	n	Negative	Positive	*P*	Low	High	*P*
Age (years)	61			1.000			0.412
< 50	11	2	9		7	4	
≥ 50	50	9	41		25	25	
Pathological type	61			0.778			0.134
Serous	49	8	41		24	6	
Other	26	3	9		3	9	
FIGO stage	61			0.139			0.210
I–II	24	7	17		13	11	
III–IV	37	4	33		14	23	
Differentiation	61			0.288			0.080
Well/moderate	22	6	16		13	9	
Poor	39	5	34		14	25	
Lymphatic	48			0.334			0.424
No	35	9	26		18	17	
Yes	13	1	12		5	8	

### High BCL9 expression was associated with a poor prognosis in ovarian cancer patients

**Fig. 2:**
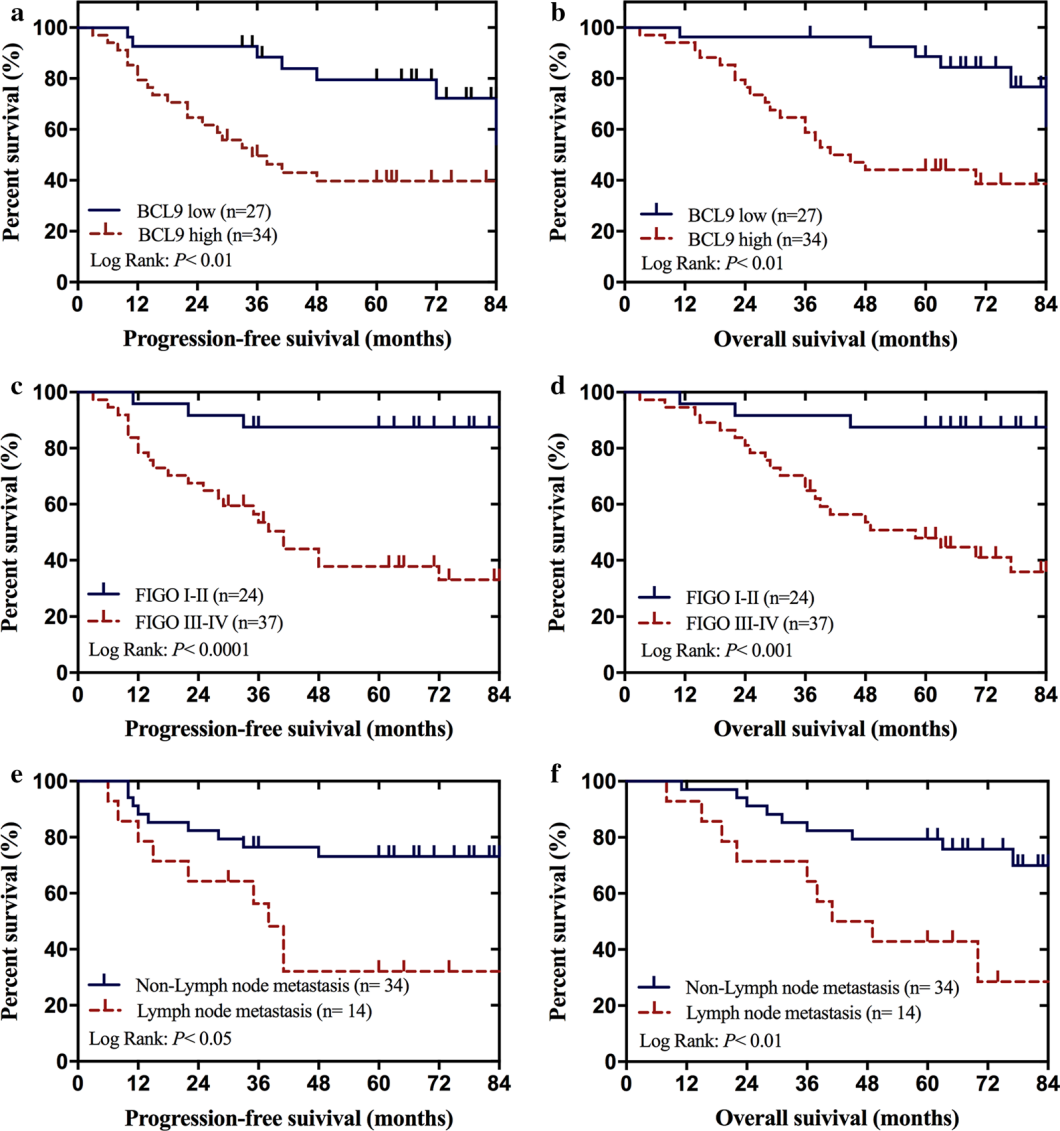
Kaplan–Meier curves of progression-free survival and overall survival in patients with epithelial ovarian cancer. **a**, **b** Correlation between BCL9 expression and progression-free survival and overall survival. **c**, **d** Correlation between clinical stage and progression-free survival and overall survival. **e**, **f** Correlation between lymph node metastasis and progression-free survival and overall survival. All *P *< 0.05

We found that the level of BCL9 expression, Federation International of Gynecology and Obstetrics (FIGO) stage, and lymph node metastasis were associated with progression-free survival (PFS) and overall survival (OS) in patients, based on the log-rank test (Fig. 2). We then performed Cox regression analysis to assess the relationship between the level of BCL9 expression, FIGO stage, lymph node metastasis, and survival in patients with ovarian cancer. Both the univariate and multivariate regression analyses indicated that FIGO stage and BCL9 expression were significantly associated with PFS (Table 3) and OS (Table 4), suggesting that they can be used as biomarkers to independently predict prognosis in ovarian cancer patients.

**Table 3:** Cox regression analysis of progression-free survival in epithelial ovarian cancer patients

Variable	Univariate analysis			Multivariate analysis		
	HR	Hazard ratio 95% confidence interval	*P*	HR	Hazard ratio 95% confidence interval	*P*
Age (< 50 years vs. ≥ 50 years)	1.115	0.452–2.749	0.814	0.59	0.185–1.880	0.372
FIGO stage (I–II vs. III–IV)	5.684	1.964–16.456	0.001**	6.655	1.769–25.040	0.005**
BCL9 expression (low vs. high)	3.382	1.432–7.988	0.005**	3.721	1.230–11.253	0.020*
Lymph node metastasis (yes vs. no)	2.628	1.082–6.387	0.033*	1.186	0.424–3.316	0.746

**P < 0.01. *P < 0.05

**Table 4:** Cox’s regression analysis of OS of ovarian epithelial cancer

Variables	Univariate analysis			Multivariate analysis		
	HR	95% CI of HR	P-value	HR	95% CI of HR	P-value
Age (< 50 vs. ≥ 50)	0.944	0.381–2.34	0.902	0.384	0.119–1.244	0.111
FIGO stage (I–II vs. III–IV)	5.188	1.788–15.058	0.002**	6.655	1.628–27.205	0.008**
BCL9 expression (low vs. high)	4.126	1.657–10.273	0.002**	4.741	1.450–15.495	0.010*
Lymph node metastasis (yes vs. no)	2.961	1.198–7.32	0.019*	1.419	0.504–3.994	0.508

**P < 0.01. *P < 0.05

### BCL9-siRNA interfered with BCL9 expression in CaoV3 and ES-2 ovarian cancer cell lines

**Fig. 3:**
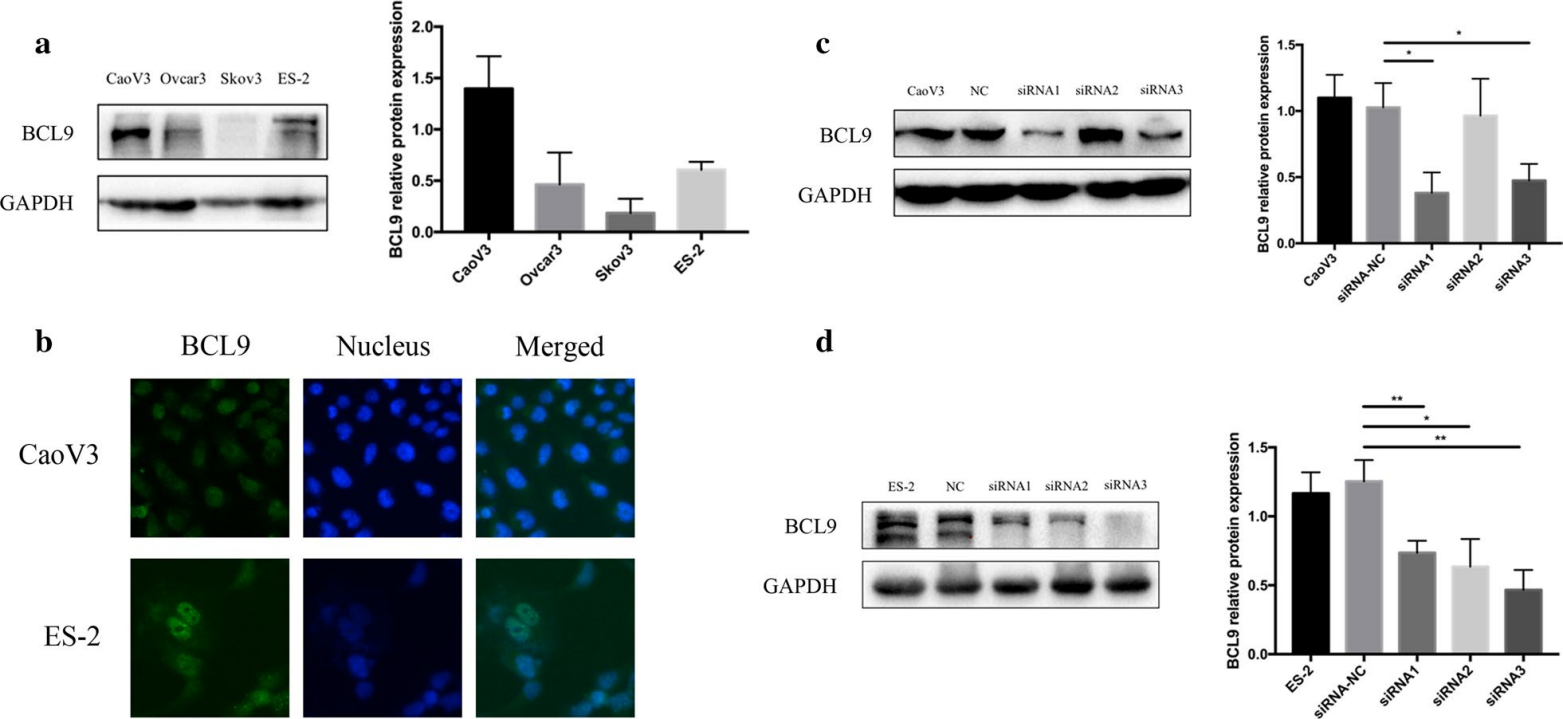
BCL9-siRNA effectively interfered with the expression of BCL9 in CaoV3 and ES-2 ovarian cancer cell lines. **a** Western blot showed that BCL9 expression was relatively high in CaoV3 and ES-2 ovarian cancer cell lines. **b** The results of the immunocytofluorescence test suggested that BCL9 was located in the nucleus and cytoplasm of CaoV3 and ES-2 cells, but mainly in the nucleus. **c**, **d** Western blot showed that BCL9 protein expression levels significantly decreased in BCL9-siRNA-transfected CaoV3 and ES-2 ovarian cancer cell lines (*P < 0.05, **P < 0.01)

We used Western blot to detect BCL9 expression in CaoV3, Ovcar3, Skov3, and ES-2 ovarian cancer cell lines. The results showed that BCL9 expression was relatively high in CaoV3 and ES-2 cells (Fig. 3a). The results of the immunocytofluorescence test suggested that BCL9 was located in the nucleus and cytoplasm of CaoV3 and ES-2 cells, but mainly in the nucleus (Fig. 3b). BCL9-siRNA was transfected into CaoV3 and ES-2 by lipofectamine, and BCL9 expression was detected using Western blot. The results showed that BCL9 protein expression in CaoV3 and ES-2 cells significantly decreased after siRNA interference (Fig. 3c, d). In all the functional relevance and western blot studies, we only transferred siRNA3 into both Caov3 and ES-2 cell lines to decrease the expression of BCL9.

### Decrease in BCL9 expression inhibited cell proliferation, which may be attributable to increases in the BAX/BCL2 ratio and apoptosis of ovarian cancer cells

**Fig. 4:**
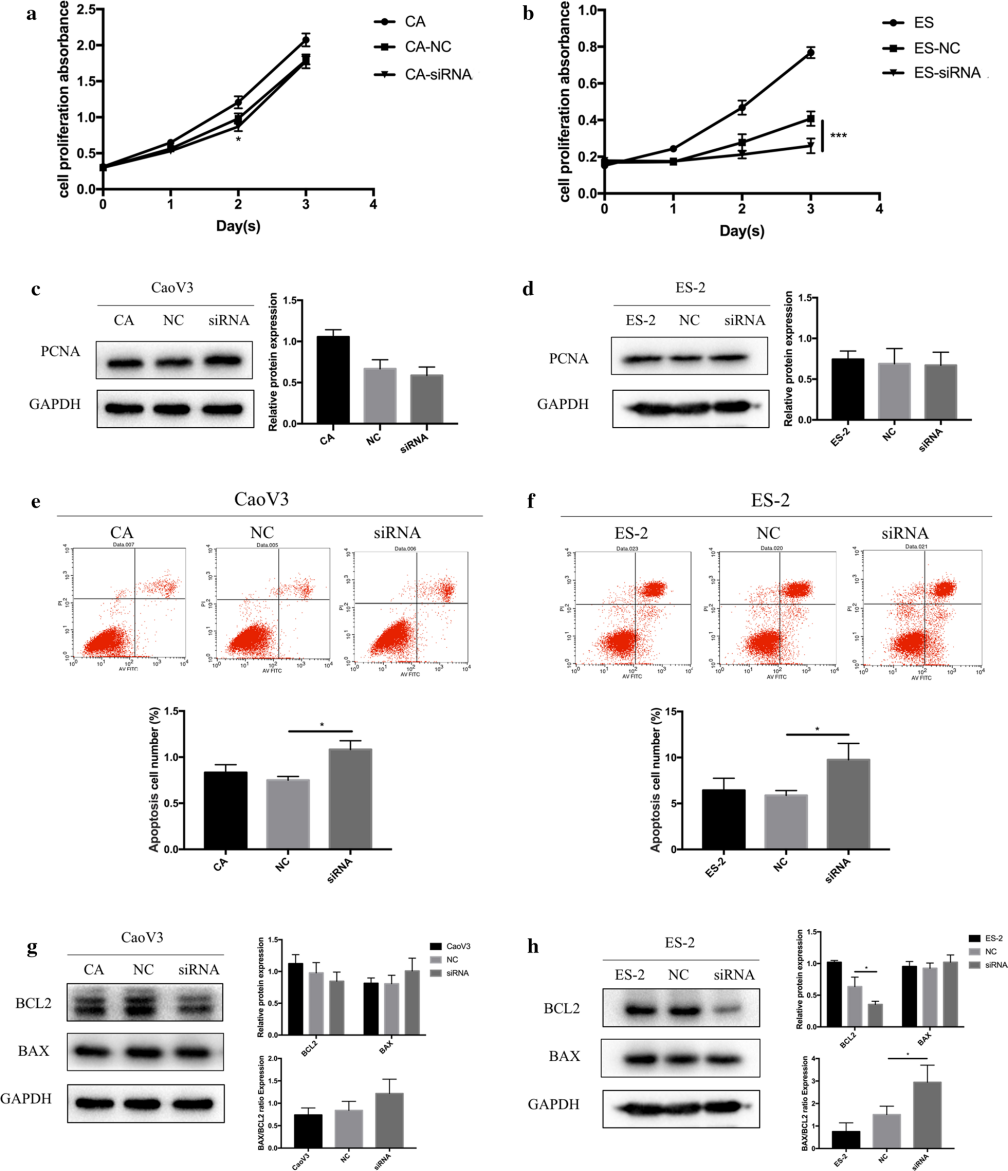
BCL9 may promote apoptosis and inhibit the proliferation of ovarian cancer cells by increasing the BAX/BCL2 expression ratio. **a** In CaoV3 cells, the maximum inhibition rate was observed 48 h after the inhibition of BCL9 expression, and the difference was statistically significant (*P < 0.05). **b** In ovarian ES-2 clear cell carcinoma, the maximum inhibition rate occurred 72 h after the inhibition of BCL9 expression (***P < 0.001). **c**, **d** No significant change in PCNA expression was detected by Western blot. **e**, **f** After inhibiting the expression of BCL9, the proportion of early apoptotic ovarian cancer cells increased (*P < 0.05). **g**, **h** After inhibiting the expression of BCL9, the expression of BCL2 decreased, the expression of BAX was unchanged, and the BAX/BCL2 expression ratio significantly increased. Significant differences from ES-2 cells were observed (*P < 0.05), with no significant differences from CaoV3 cells

To investigate the effect of BCL9 on the proliferation and apoptosis of ovarian cancer cell lines in vitro, we used the MTT assay to detect changes in the proliferation of ovarian cancer cells after decreasing BCL9 expression. The MTT results showed that when the expression of BCL9 decreased, the viability of ovarian cancer cells in both groups significantly decreased (Fig. 4a, b). The reduction of ES-2 cell activity was greater than the reduction of CaoV3 cell activity. No significant changes in the expression of proliferating cell nuclear antigen (PCNA) were observed (Fig. 4c, d). Flow cytometry was used to detect cell apoptosis. The results showed that the rate of the early apoptosis of ovarian cancer cells significantly increased after inhibiting the expression of BCL9 (Fig. 4e, f). Western blot showed that the expression of apoptosis-promoting BCL2 protein decreased, with no changes in the expression of apoptosis-related BAX protein. The BAX/BCL2 expression ratio significantly increased (Fig. 4g, h). This indicates that the decrease in BCL9 expression inhibited ovarian cancer cell proliferation by promoting the apoptosis of ovarian cancer cells by increasing the BAX/BCL2 expression ratio.

### Decrease in BCL9 expression inhibited the invasion and migration of ovarian cancer cells and inhibited MMP2 and MMP9 expression of ES-2 cell line

**Fig. 5:**
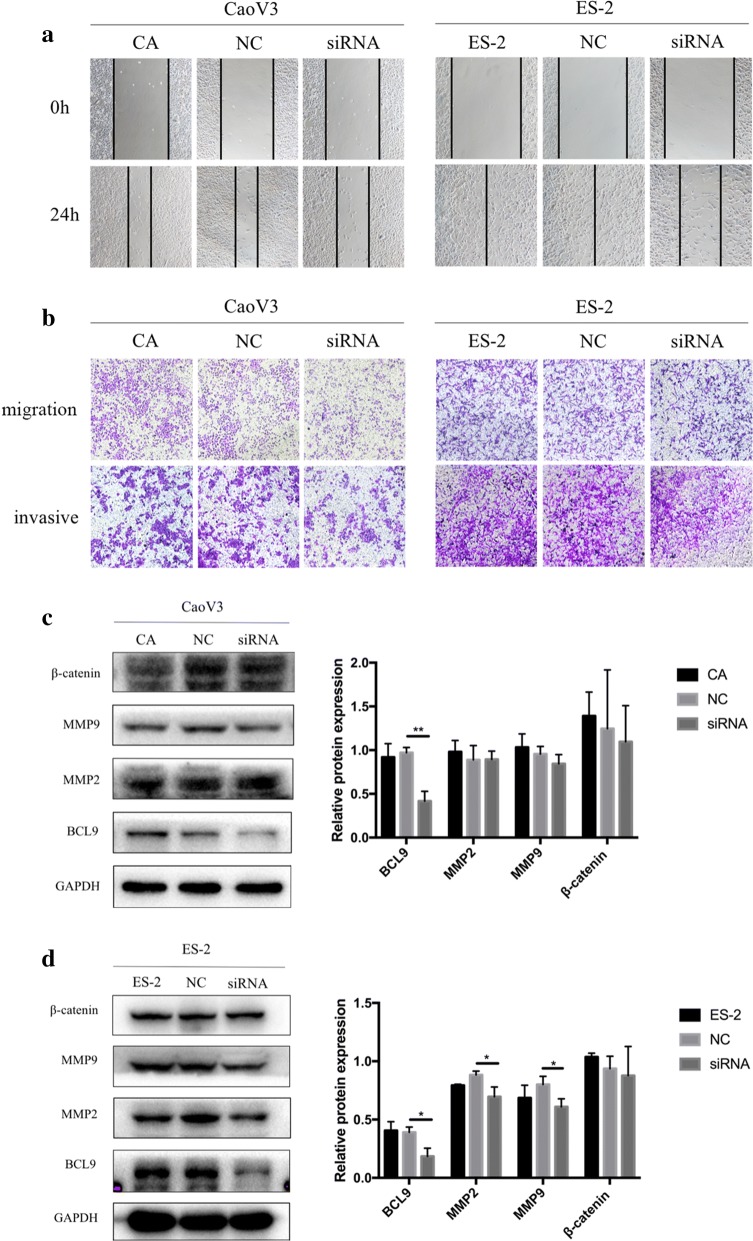
Inhibition of BCL9 may inhibit the invasion and migration of ovarian cancer cells by inhibiting MMP2 and MMP9. **a** The results of the scratch test showed that the migration distance of ovarian cancer cells in the low BCL9 expression group after 24 h was significantly less than in the control group. **b** The transwell test results showed that the cell migration ability of ovarian cancer cells with low BCL9 expression decreased within 24 h compared with the control group, and the cell invasion ability decreased within 48 h. **c**, **d** After inhibiting BCL9 expression, the expression of MMP2 and MMP9 significantly decreased in ES-2 cells (*P < 0.05, **P < 0.01), but BCL9 expression did not affect β-catenin expression

To analyze the effect of BCL9 expression on the invasion and migration of ovarian cancer cells, we decreased BCL9 expression and detected changes in related proteins. The results of both the scratch test and transwell test showed that ovarian cancer cells with low BCL9 expression had lower migration and invasion ability than the control group. The degree of inhibition of ES-2 cells was higher than CaoV3 cells (Fig. 5a, b). We then examined the expression of matrix metalloproteinase 2 (MMP2) and MMP9 proteins that promote cell invasion and migration. Western blot showed that MMP2 and MMP9 expression significantly decreased only in ovarian ES-2 clear cancer cells (Fig. 5c), with no significant difference in ovarian CaoV3 serous carcinoma cells (Fig. 5d). These results suggested that low BCL9 expression inhibited the invasion and migration of ovarian cancer cells.

**Fig. 6:**
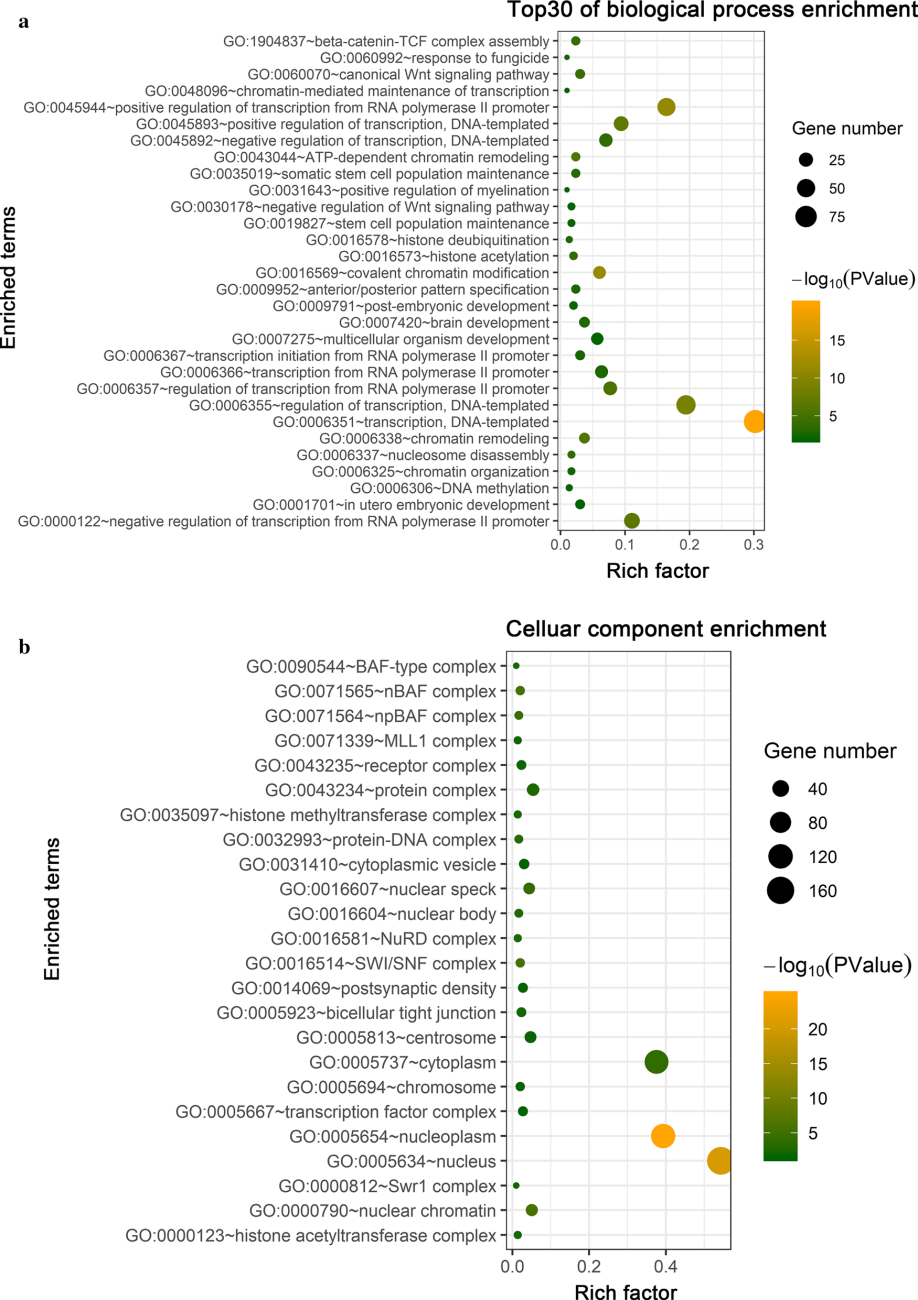
Using the Cbioportal and DAVID databases to analysis the pathway function of BCL9 and its associated molecular enrichment. **a** Biological processes that involve BCL9-related molecules. **b** Molecular function of BCL9-related molecules. The ordinate shows the name of the functional area. The abscissa shows the percentage of the corresponding part of the network as a whole. The bubble size indicates the number of genes that are located in the pathway or function. Colors indicate enriched *P* values. Deeper colors indicate more significant results

**Fig. 7:**
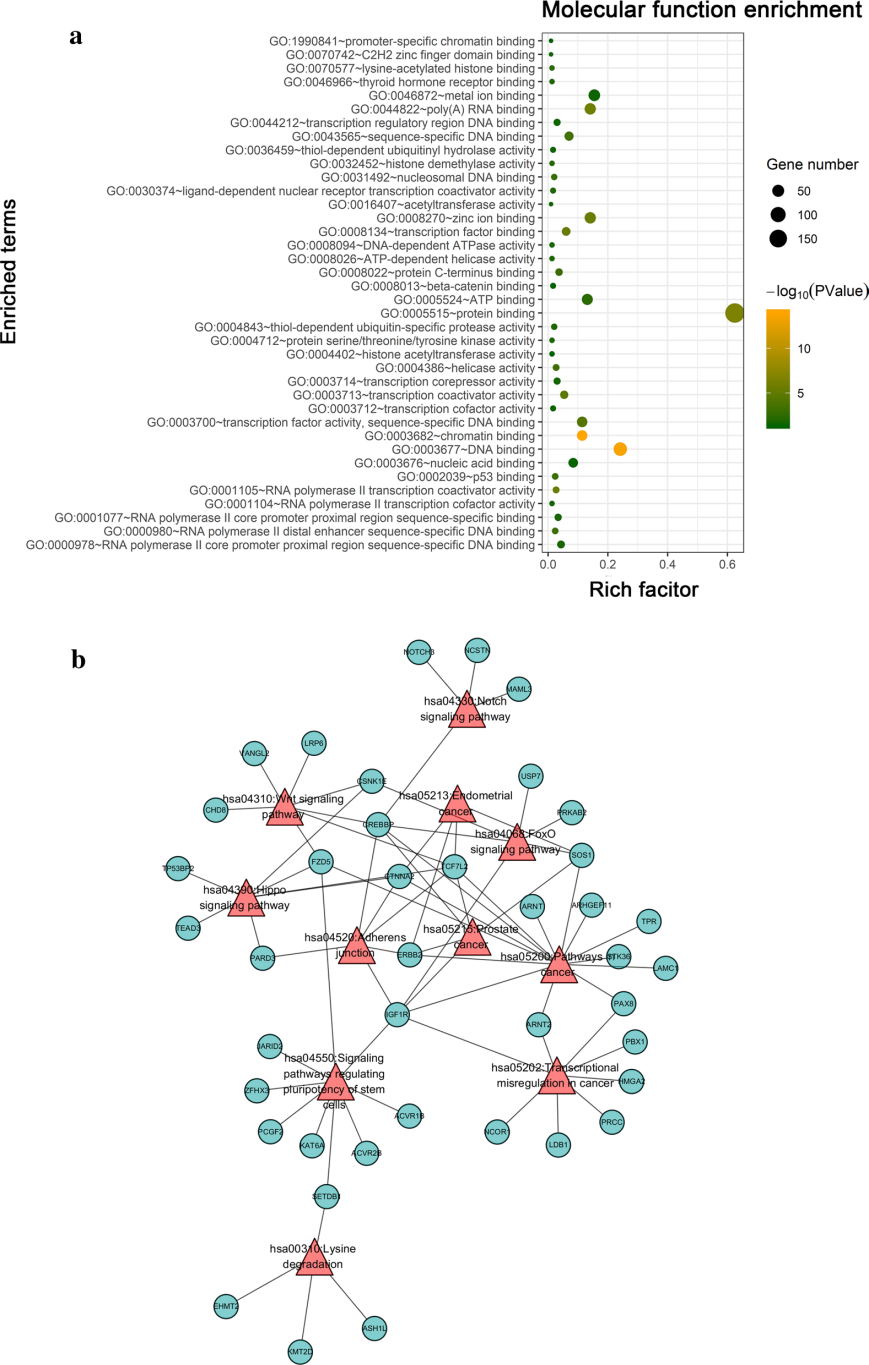
Enriched locations of the proteins encoded by BCL9-related molecules and the pathway regulatory network map of BCL9-related molecules. **a** Enriched locations of the proteins encoded by BCL9 related molecules. **b** Pathway regulation network map of BCL9-related molecules. The green circle represents the gene. The red triangle represents the enriched pathway

**Fig. 8:**
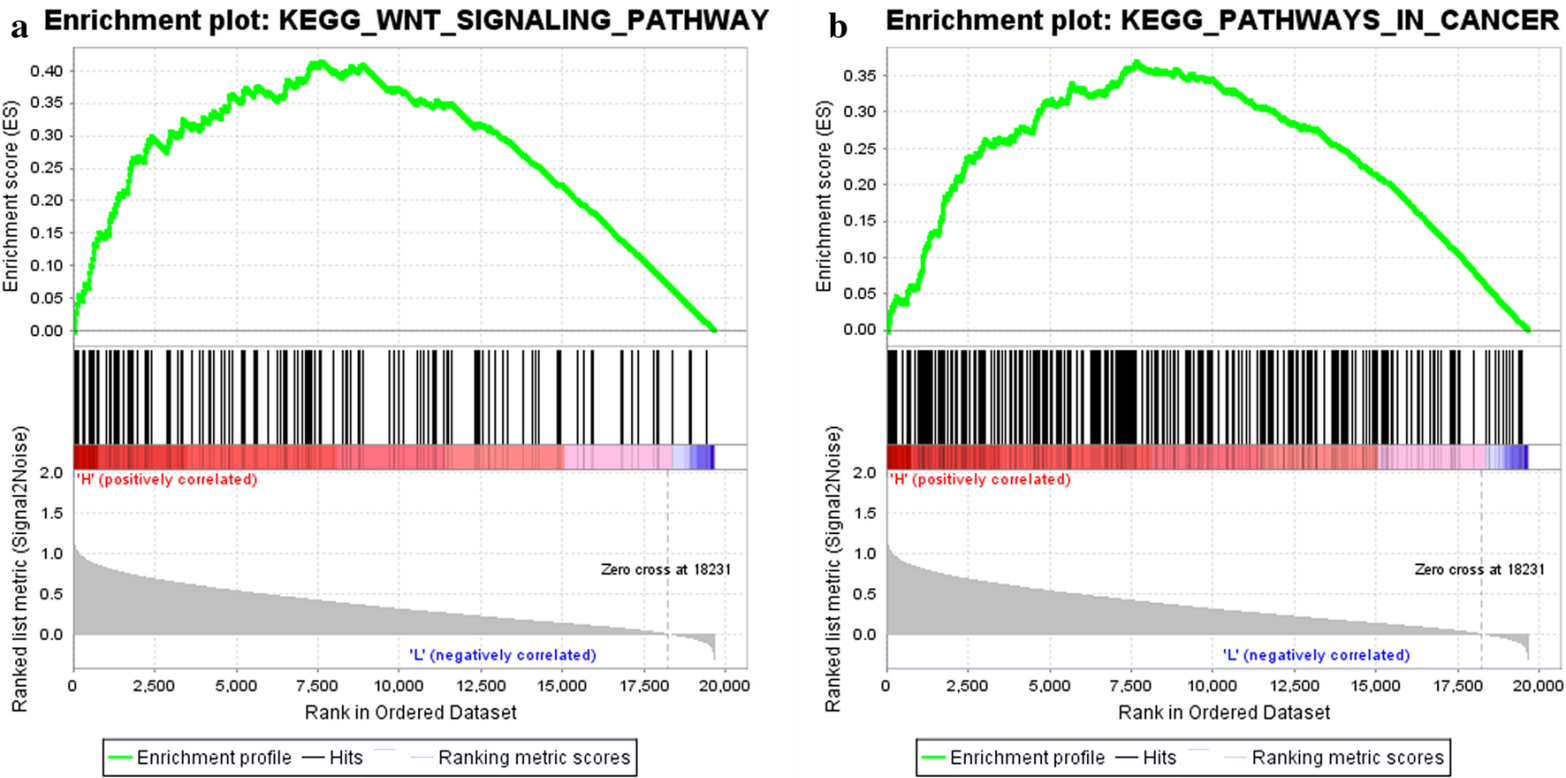
The GSEA indicated that samples with high BCL9 expression were also significantly enriched in the Wnt signaling pathway and the pathways in cancer. **a** Wnt signaling pathway. **b** Pathways in cancer

Function and pathway enrichment analyses of BCL9-related molecules were performed using the Cbioportal and DAVID databases. The top 30 gene ontology (GO) terms were visualized using the “ggplot2” R software package. The genes were mainly found in the canonical Wnt signaling pathway (GO: 0060070) and β-catenin-TCF complex assembly (GO: 1904837) (Fig. 6a). The molecular functions involved DNA binding (GO: 0003677), transcription factor binding (GO: 0008134), and p53 binding (GO: 0002039) (Fig. 6b). The proteins that are encoded by these related molecules are located in the cytoplasm (GO: 0005737), nucleoplasm (GO: 0005654) and nucleus (GO: 0005634) (Fig. 7a). The pathway regulatory network map indicated that the gene pathways were pathways in cancer (hsa05200), Wnt signaling pathway (hsa04310), and adherens junction and other signaling pathways (hsa04520) (Fig. 7b). The GSEA indicated that samples with high BCL9 expression were also significantly enriched in the Wnt signaling pathway and the pathways in cancer (Fig. 8a, b). These results were consistent with the DAVID database. Therefore, BCL9 appears to affect cell proliferation by regulating the Wnt signaling pathway and other tumor-related signaling pathways to promote the occurrence and development of ovarian cancer. We used western blot experiments to verify the pathways involved. The results suggested that after decreasing BCL9 expression, β-catenin expression was not changed (Fig. 5c, d).

## Discussion

The classic Wnt signaling pathway is composed of receptor-mediated signal transduction systems that are highly evolutionarily conserved and strictly regulated. Abnormal activation of the Wnt signaling pathway is closely related to the occurrence of epithelial cancers, such as colorectal cancer and hematologic malignancies (e.g., multiple myeloma) [11–14]. In the classic Wnt signaling pathway, the stability of β-catenin and regulation of its concentration are crucially important. BCL9 is mainly an adapter protein and newly identified member of the classic Wnt signaling pathway. BCL9 recruits and binds other cofactors to the β-catenin/TCF complex in the nucleus. BCL9 has two main functional domains: HD1 and HD2. The HD1 domain specifically binds to the N-terminus of β-catenin. The HD2 domain binds to the Pygo PHD region, thus indicating that BCL9 serves as a bridge to β-catenin and Pygo [7]. The aforementioned complex and other transcriptional cofactors that bind to the transcriptional activation region of the C-terminus of β-catenin form a complete complex. This complex interacts with TCF/LEF, which binds to the WRE sequence and subsequently promotes the dissociation of transcription repressors on TCF/LEF [15, 16]. This process initiates the transcription of downstream genes (e.g., CD44, VEGF, c-Myc, and cyclin D1) [17], leading to the proliferation of cancer cells and occurrence and development of tumors. These findings indicate that BCL9 plays an important role in the Wnt pathway.

BCL9 promotes the proliferation, invasion, migration, angiogenesis, and epithelial–mesenchymal transition of colon cancer cells and multiple myeloma cells, but it does not affect β-catenin protein expression. The knockdown of BCL9 expression increased survival rate in a nude mouse cancer cell xenograft model, reduced vascular formation in transplanted tumors, and inhibited tumor metastasis [17]. High BCL9 expression was associated with a poor prognosis in colon cancer patients [18]. Brown et al. [19] reported that BCL9 was overexpressed in malignant adrenal cortical tumor tissues and promoted the proliferation of adrenal cortical tumor cells. He et al. [20] reported that the upregulation of BCL9 was associated with early diagnosis and the degree of malignancy of prostate cancer, revealed by immunohistochemistry, but BCL9 was not an independent biomarker for predicting the non-biochemical recurrence survival rate in prostate cancer patients. Hyeon et al. [21] used immunohistochemistry and found that the overexpression of BCL9 in hepatocellular carcinoma tissue was significantly associated with high Edmondson grade, microvascular infiltration, and the intrahepatic metastasis of hepatocellular carcinoma, which had adverse effects on both disease-free survival and disease-specific survival. These findings suggest that BCL9 expression may be a biomarker of low disease-free survival in hepatocellular carcinoma patients after radical hepatectomy.

In the present study, we detected high BCL9 expression in ovarian cancer tissues. High BCL9 expression was associated with 5-year PFS and 5-year OS in ovarian cancer patients, suggesting that BCL9 expression and may serve as an independent biomarker for predicting survival and prognosis in ovarian cancer patients. The B lymphocyte-2 gene (B-cell lymphoma-2 [BCL-2]) is one of the most extensively investigated oncogenes in cell apoptosis research. The BCL2 protein family can be divided according to function and structure into anti-apoptotic BCL2 proteins (BCL2, MCL1, and BCLxL) and pro-apoptotic effectors (BAK, BAX, and BOK) [22]. We decreased BCL9 expression in the ovarian CaoV3 serous cancer cell line and ovarian ES-2 clear cancer cell line, which inhibited BCL2 expression and increased the BAX/BCL2 expression ratio, promoted cell apoptosis, and inhibited the proliferation of ovarian cancer cells. Therefore, we speculate that BCL9 affects the proliferative capacity of ovarian cancer cells by promoting the apoptosis of ovarian cancer cells, thus promoting the development of ovarian cancer. MMP2 and MMP9 are members of the MMP family. Matrix metalloproteinases are matrix-hydrolyzing enzymes that dissolve collagen. The upregulation of MMPs is positively correlated with tumor progression, metastasis, and poor prognosis [23, 24]. The invasion and migration ability of CaoV3 and ES-2 cells were inhibited after we interfered with BCL9 expression. Low BCL9 expression also downregulated the expression of MMP2 and MMP9 in ES-2 cells, suggesting that BCL9 might dissolve the extracellular matrix of ovarian cancer cells by promoting the expression of MMP2 and MMP9, further promoting the invasion and metastasis of ovarian cancer cells. However, this was not applicable to the CaoV3, which suggested that BCL9 played different degree of role in different cell lines of ovarian cancer. Based on the literature and bioinformatics analyses, BCL9 appears to promote the development of ovarian cancer by regulating the Wnt signaling pathway. Therefore, we detected the expression of β-catenin when the expression of BCL9 was downregulated. The results suggested that BCL9 does not affect the expression of β-catenin in either of the ovarian cancer cell lines, which is consistent with research on multiple myeloma and colon cancer cells [17].

Ovarian cancer is a common malignant tumor in female genital organs. Ovarian cancer has the third highest incidence and highest mortality rate. More than 75% of ovarian cancer patients are diagnosed at the middle or advanced stage. Clinical remission can be achieved in patients with advanced ovarian cancer after surgery and adjuvant chemotherapy, but serious adverse reactions, drug resistance, relapse, and low overall survival have continued the search for more effective treatments. Molecular targeted therapy for tumors uses small-molecule compounds, monoclonal antibodies, and peptides to interfere with molecules that are involved in the occurrence and development of tumors to induce changes at the molecular level and exert anti-tumor effects. Molecular targeted therapy plays a pivotal role in the treatment of malignant tumors, but the development of molecularly targeted drugs for ovarian cancer, including monoclonal antibodies, signaling pathway inhibitors, angiogenesis inhibitors, and gene therapy, has been slow. Most such molecular therapies are still undergoing clinical trials, with no confirmation of efficacy. Bevacizumab and three PARP inhibitors (i.e., olaparib, rucaparib, and nilapani) are the only molecularly targeted drugs that are currently approved for the treatment of advanced ovarian cancer in the United States and other countries, but their clinical application has some limitations. A treatment that targets mir-30-5p and inhibits BCL9 was shown to reduce tumor burden and metastatic potential in a human multiple myeloma cell xenograft model in mice, which affects the orthopedic diseases of mice irreversibly [25, 26]. In addition to miR-30-5p, miR-1301, miR-30a, miR-218, and miR-30c also downregulate the expression of BCL9 in different cancer tissues [25–29]; among these, miR-30c-2 worked in ovarian cancer [30].

Additionally, the development of small-molecule inhibitor drugs that target the oncogene interaction interface has potential. BCL9 acts mainly as a connexin that attracts other cofactors in the Wnt pathway to form a complex with β-catenin/TCF to activate the expression of various oncogenes. If a small molecule can destroy the interaction interface of proteins, such as β-catenin–BCL9–TCF, to prevent them from forming a complex, then this may inhibit the Wnt signaling pathway in tumor cells and prevent the occurrence and development of tumors. Kawamoto et al. [31] successfully synthesized a peptide based on BCL9 in 2009, which could completely inhibit the binding of β-catenin to BCL9 protein. Fan et al. [32] reported that sex-determining region Y-Box 7 (SOX7) binds to β-catenin, competing with BCL9 and inhibiting β-catenin-mediated transcription by disrupting the β-catenin/BCL9 interaction. Recently, John et al. [33] synthesized a small-molecule inhibitor with a 1,4-dibenzyl piperazine scaffold structure that has actions on the β-catenin/BCL9 protein–protein interaction interface. The binding mode was characterized by structure–activity relationships and site-directed mutagenesis, indicating high selectivity and strong interference ability. These authors also found that this series of inhibitors selectively inhibited classic Wnt signaling and inhibited the growth of Wnt/β-catenin-dependent cancer cells.

## Conclusions

In conclusion, BCL9 is highly expressed in EOC tissues and may be an independent biomarker for predicting prognosis in ovarian cancer patients. Low BCL9 expression can promote cell apoptosis, inhibit the proliferation, invasion, and migration of ovarian cancer cells. BCL9 promotes the occurrence and development of ovarian cancer. Although the precise mechanism of action remains to be determined, small-molecule anti-tumor drugs that target the β-catenin/BCL9 interface may be promising for the treatment of ovarian cancer. These findings provide an important experimental basis for the biological function of BCL9 in the progression of EOC, which provides new ideas and possibilities for the treatment of EOC.

## Data Availability

The datasets used or analyzed during the current study are available from the corresponding author upon reasonable request.

## Abbreviations

BCL9: B-cell lymphoma 9
EOC: epithelial ovarian cancer
TCGA: The Cancer Genome Atlas
MMP2: matrix metalloproteinase 2
MMP9: matrix metalloproteinase 9
BCL2: B-cell lymphoma 2
BAX: BCL2 associated X protein
TCF: T cell factor transcriptional cofactor
LEF: lymphocyte enhancer
FIGO: International Federation of Obstetrics and Gynecology
PCNA: proliferating cell nuclear antigen
GSEA: gene set enrichment analysis
FDR: false discovery rates
PFS: progression-free survival
OS: overall survival
VEGF: vascular endothelial growth factor
MCL1: myeloid cell leukemia-1
